# The evaluation of an interactive web-based Pulmonary Rehabilitation programme: protocol for the WEB SPACE for COPD feasibility study

**DOI:** 10.1136/bmjopen-2015-008055

**Published:** 2015-08-25

**Authors:** Emma Chaplin, Stacey Hewitt, Lindsay Apps, Kelly Edwards, Chris Brough, Aga Glab, John Bankart, Ruth Jacobs, Sally Boyce, Johanna Williams, Sally Singh

**Affiliations:** 1 Leicester Respiratory Biomedical Research Unit, Department of Respiratory Medicine, Centre for Exercise and Rehabilitation Science, Glenfield Hospital, University Hospitals of Leicester, Leicester, UK; 2 Department of Primary Care and Health Sciences, Keele University, Keele, UK; 3 Health Sciences Research Institute, Medical School, University of Warwick, Coventry, UK; 4 School of Sport, Exercise and Health Sciences, Loughborough University, Loughborough, UK

**Keywords:** REHABILITATION MEDICINE

## Abstract

**Introduction:**

Pulmonary Rehabilitation (PR) is an evidence-based intervention that has been recommended in guidelines to be available to those who may benefit. However, not all patients with chronic obstructive pulmonary disease (COPD) have access to this service. Healthcare services have shown the need for the provision of PR in other forms to enable patient choice and service capacity. There is an increase in evidence for the use of the internet in the management of long-term conditions to provide education and promote self-management. The aim of this study is to see if an interactive web-based PR programme is a feasible alternative compared with conventional PR.

**Methods and analysis:**

This is a feasibility study designed to evaluate the efficacy of providing a web-based PR programme to improve patients exercise capacity, quality of life and promote self-management in patients with moderate to severe COPD compared with conventional PR programmes. Eligible patients will be randomly allocated to receive either the web-based programme or conventional rehabilitation programme for 7 weeks using an internet-based randomisation system. Participants will be recruited from PR assessments, primary care and community rehabilitation programmes. Those randomised to the web-based programme work through the website which contains all the information that the patients receive in the PR classes. They receive weekly phone calls by a professional to help progress through the course on line. The outcome measures will be recruitment rates and eligibility as well as that standard for a PR assessment including measures of exercise capacity, quality of life questionnaires and physical activity.

**Ethics and dissemination:**

The research ethics committee for Northampton has provided ethical approval for the conduct of the study. The results of the study will be disseminated through appropriate conference presentations and peer reviewed journals.

**Trial registration number:**

ISRCTN03142263.

**Strengths and limitations of this study**

- The study concentrates on the feasibility of an interactive web-based Pulmonary Rehabilitation (PR) programme (SPACE: Self-management Program of Activity, Coping and Education for chronic obstructive pulmonary disease). It will provide data on recruitment to enable an accurate estimation of sample size for a planned randomised controlled trial.
- Much of the success depends on the eligibility of the patients, that is, web literacy and access to the internet.
- The study will compare a variety of clinical outcomes between a web-based and a conventional rehabilitation PR programme in order to test the various components of the intervention.

## Introduction

It is estimated that one in four people will develop chronic obstructive pulmonary disease (COPD) in their lifetime^1^ and it is the fourth leading cause of death in the UK. COPD is characterised by a progressive deterioration of debilitating symptoms and increasingly frequent exacerbations. Pulmonary Rehabilitation (PR) has been proven to be effective in improving quality of life, psychological functioning and physical activity and national guidelines recommend that PR should be available to all those who may benefit.^2^ However a survey by the British Lung foundation has found that fewer than 10% of people with COPD have access to such a service.^3^

The standard provision of PR is a supervised package of exercise and education, usually twice a week for a minimum of 6 weeks supported by a home exercise programme. However, some patients find visiting a hospital or community facility very difficult and many programmes report dropout rates as high as 50%. A web-based PR programme has the potential to be a novel and effective approach to increasing patient choice in the mode of delivery and setting of rehabilitation (especially to those patients who decline the offer of conventional PR) while simultaneously increasing the capacity of PR.

There is a growing evidence base for the use of the internet in the management of many chronic conditions in areas as diverse as the management of diabetes, Parkinson's disease, depression, rheumatoid arthritis, asthma, chronic pain and epilepsy.^4–10^ In COPD, Nguyen *et al*^11^ published pilot data on an online dyspnoea self-management programme which had comparable outcomes to a face to face programme but was stopped early due to multiple technical challenges with the online programme.

Our institution has recently pioneered the development of ‘ACTIVATE ***YOUR*** HEART’ (AYH)^12^ which is an interactive web-based cardiac rehabilitation (CR) programme and has proved very popular with patients, with the recruitment of 80 patients far exceeding the initial target of 25. Brough *et al* (2014) reported a significant improvement in exercise capacity and quality of life in patients that completed the web-based programme. Following the success of AYH, we have developed a prototype website based on the educational content of the ‘SPACE for COPD’ self-management workbook. SPACE (Self-management programme of Activity, Coping and Education) for COPD 13 is a structured programme of exercise, education and psychosocial support which has been developed by our institution as a collaboration between experts, patients and carers and has been awarded a Crystal Mark for Clarity by the Plain English Campaign.

The aim of this feasibility study therefore is to provide quantitative, qualitative, economic and technical data which will inform and refine the subsequent design of a randomised controlled trial (RCT) which will compare web-based and conventional PR across a range of clinical and health economic outcomes.

The objectives for the study are:

1. To gather information regarding the recruitment rate of patients who are eligible and willing to be randomised to either the web-based or the conventional rehabilitation programme, and to monitor retention and drop out through all stages of the programmes.
2. To perform a pilot study which will compare a variety of clinical outcomes between an web-based and a conventional rehabilitation PR programme in order to test out the various components of the intervention and identify any technical or other difficulties that may be inherent in the delivery of an web-based PR programme.
3. To collect data regarding the primary outcome measure (change in exercise capacity as measured by the incremental shuttle walking test) which will allow a calculation of sample size to be estimated in the eventual trial.
4. To explore patients’ experiences of participating in the rehabilitation programmes in order to identify barriers and facilitators to recruitment and retention in the subsequent RCT and to identify any further changes needed in the design of the website.
5. To design and pilot a suitable patient cost questionnaire to be used in the proposed cost-effectiveness economic evaluation in the subsequent RCT.

## Methods and analysis

### Trial design and registration

This is a feasibility study to inform the design of a RCT. Patients are randomised to either conventional rehabilitation programme as is standard at their referred site or the web-based PR programme (SPACE for COPD).

The trial is registered on the ISRCTN website. The trial was registered prospectively by the local CLRN. The final authorisation came through the day after the first patient had been recruited.

### Participants

#### Eligibility criteria for participants

Eligible participants are those with an established diagnosis of COPD defined as a forced expiratory volume in 1 s (FEV1), post-bronchodilation of <80% and a predicted ratio of FEV1 to forced vital capacity of 0.70 and a Medical Research Council (MRC) dyspnoea score of between 2 and 5. Patients must be willing to partake in either arm of the study. Access to the internet for more than 3 months, the ability to navigate around a variety of websites (eg, uses online shopping or banking websites) and regularly use email is required. Patients must also be able to read and write in English.

Patients are excluded from participating in the trial if they are unable to participate in the exercise component of the rehabilitation programme due to other comorbidities. Eligible patients must be willing and able to take part in the web-based programme. Participation in PR in the previous 12 months would also exclude the patient.

#### Setting

Participants to the study will be primarily recruited from those patients that have been referred for PR at University Hospitals of Leicester (UHL) NHS Trust. Recruitment will also be directly from primary care and Rehabilitation services within Leicester Partnership Trust (LPT). All potentially suitable participants will receive a patient information sheet about the study, an invitation letter on behalf of the study team as well as their initial letter confirming their referral to PR, as is standard for the LPT service. Potentially eligible participants will also be identified from the research participant database of the Leicester Respiratory Biomedical Research Unit and PR Database.

### Trial interventions

#### Intervention development

A preprotocol award from the NIHR East Midlands Research Design Service (RDS) enabled us to conduct a focus group with current and ex-PR patients to gain feedback on the prototype website, with particular regard to features that increase interactivity and usability for service users as well as addressing any concerns such as data security. The model for the interactive web-based PR programme has been based on recent, similar work by our institution on the ‘ACTIVATE ***YOUR*** HEART’ web-based CR programme and the previously successful SPACE manual.^13^ Three patients who took part in AYH also took part in the focus group. Information from the focus group has enabled the project team to work with the web-designers to ensure that the final design of the website is in line with these preferences, ideas and feedback. The website has undergone practical ‘road-testing’ by members of the focus group and other members of the departmental patient and public involvement (PPI) group to ensure that participants can access the website and navigate easily around the site.

#### Intervention group—web-based PR programme

Following randomisation to the intervention group, patients will be given an appointment to attend an initial session that will introduce the web-based PR programme. At this initial session, the website will be introduced by a PR specialist who is trained in the techniques of motivational interviewing as well as being an expert in PR. Participants will be given a password protected secure login to the website as well as written instructions on website navigation.

The rehabilitation specialist will signpost the patient to all the relevant sections on the website for getting started with the programme as well as going through the home exercise programme and goal setting. There is also an individualised webpage featuring a personalised action plan designed to assist in the management of exacerbations which will be completed by the rehabilitation specialist in conjunction with the patient.

As in conventional PR, patients will be encouraged to exercise on a daily basis at home and record their progress in the online exercise diary section. Throughout the duration of the web-based programme the rehabilitation specialist will be able to review the patient's progress online and communicate with the patient via email or telephone depending on patient preference. There will be weekly contact between the patient and the rehabilitation specialist to ensure that patients are helped to progress their exercise programme appropriately and to answer any queries that arise.

The educational content of the web-based programme is based on the ‘SPACE for COPD’ manual. Patients on the intervention arm of the study will be allowed to work through the website content at their own pace, however, certain milestones need to be completed or achieved before further content can be accessed in order to ensure appropriate progress through the programme. We anticipate from our work with a comparable CR web-based programme ‘ACTIVATE ***YOUR*** HEART’, that it will take approximately 6 weeks to work through the online programme. Patients will then be given an appointment to come into the hospital to attend a discharge appointment once they have completed the programme. The patient is offered a course of conventional rehabilitation classes if they feel it would be more beneficial.

#### Standard care group—conventional PR programme

Standard care for patients referred for PR at UHL is hospital-based rehabilitation and community-based for those referred to LPT. The PR programme at UHL has been running for nearly 20 years and is a well-established programme with a strong evidence base.^14^ ^15^ Patients randomised to standard care will start conventional rehabilitation according to the standard care at their referred site on the next available date.

Conventional PR programmes consist of twice weekly sessions each lasting 2 h which are divided into an hour for exercise training and an hour for an education session covering a variety of relevant self-management topics. The exercise training consists of endurance based walking, static cycling and strength training with weights. The programme is run by PR specialists who progress people through the exercise programme as able and appropriate. Patients are encouraged to also complete a home exercise programme on the days when they do not attend rehabilitation classes and to fill in an exercise diary. The educational sessions are conducted as group sessions and delivered by experts in their field. There is plenty of opportunity for discussion as well as questions and answers and the topics covered include medication, relaxation skills, chest clearance and breathlessness management and energy conservation.

During the period of their rehabilitation patients are given a telephone number so they can contact a member of their rehabilitation team if they have any queries or concerns.

After completing the conventional rehabilitation sessions patients will be given an appointment to attend a discharge assessment (usually approximately 6–7 weeks after starting the programme) as is standard for their rehabilitation service.

At the end of the discharge assessment all patients will be given advice on continuing their home exercise programme, regardless of group allocation.

### Outcome measures

#### Clinical measures

All the measures described below are routinely included in the clinical evaluation of our standard PR programme, with the exception of the use of activity monitors. All measures (except for activity monitors) will be completed at the time of initial assessment for PR by members of the rehabilitation team or at the consent appointment by the research team both who are experienced in these measures. The PR assessment includes information on medical history, medication and baseline demographic data. Standard measures of lung function, level of physical activity, exercise capacity, information needs and health status will be collected.

All measures will be repeated again at the discharge assessment following completion of either rehabilitation programme (usually approximately 6–7 weeks after starting the programme) and will be conducted by a research physiotherapist who will be blind to treatment group allocation.

#### Primary outcome measure—exercise capacity

The primary outcome in any eventual trial is expected to be change in exercise capacity following rehabilitation, as measured by the Incremental Shuttle Walking Test (ISWT). The ISWT is a standardised, validated test^16^ of maximal performance and is used as a tool to prescribe the home walking programme. We will estimate a sample size for the subsequent RCT from the observed changes in ISWT in the feasibility study.

The Endurance Shuttle Walking Test (ESWT) will be used to measure endurance exercise capacity and the results of the test are expressed in seconds walked.^17^

#### Physical activity

This will be assessed using physical activity monitors. The ‘Bodymedia Sensewear’ (APC Cardiovascular, UK) activity monitor is a biaxial accelerometer that can report a number of parameters including step count and energy expenditure. It will be possible to interrogate the data to identify whether individuals accumulate 30 min of moderate activity a day. Following randomisation and prior to starting either programme, all participants will be asked to wear an activity monitor every day for the next week for at least 12 h a day (seven full days). Participants will then be asked to wear the monitor again for another week following the discharge assessment.

#### Questionnaires

The majority of the questionnaires to be used in the study are part of the routine evaluation of the PR programme. Patients will be given the questionnaires to fill in at home, following the initial assessment for PR. A week before the final discharge appointment, patients will be given (for those in the standard care group) or posted (for those on the web-based programme) the questionnaires once again to fill in at home and bring back when they attend for their final discharge appointment. Any additional measures used in the study (the EQ-5D-5L, a physical activity questionnaire, COPD Anxiety Questionnaire and a patient cost questionnaire) will only be given once the patient has consented to take part in the study.

#### Health status

Two disease specific questionnaires will be employed; the self-reported Chronic Respiratory Questionnaire (CRQ-SR) which has both an initial and follow-up version depending of time of administration^18^ and the COPD Assessment Tool (CAT).^19^ Self-efficacy will be measured using the PR Adapted Index of Self-Efficacy (PRAISE).^20^These measures are all part of the standard evaluation for PR. Participants to the study will additionally be asked to fill in the generic Euro-QOL(EQ-5D-5L).^21^

#### Anxiety and depression

The Hospital Anxiety and Depression Scale (HADS) is a simple self-reported measure that gives an indication of clinically important anxiety and depression.^22^ HADS is routinely used in the clinical evaluation of PR.

Participants will also be given the self-reported COPD Anxiety Questionnaire (CAQ) to complete before and after completing either rehabilitation programme. This questionnaire is currently being validated in English from the original German version.

#### Information needs

This will be assessed using the Bristol COPD Knowledge questionnaire (BCKQ).^23^ The BCKQ is routinely used in the clinical evaluation of PR. Patients randomised to the web-based PR programme will fill one of these out on line.

#### Patient cost questionnaire

A ‘patient cost’ questionnaire^24^ will be given to all study participants following consent. This questionnaire has been adapted for the study in order to determine costs to the patient and their family of the impact of the disease as well as any treatment they have needed for their COPD. The patient cost questionnaire will be administered again as part of the questionnaires given out prior to the discharge appointment.

#### Physical activity questionnaire

A physical activity questionnaire has been developed at UHL as part of this feasibility study. PACER (the Physical Activity Everday Recall) questionnaire has been designed to capture the everyday physical activities that people do on a regular basis. It asks people to estimate how much time they have spent doing a variety of day to day activities in the past 7 days. Participants will be asked to fill in the PACER questionnaire at the end of the week spent wearing the physical activity monitor. This will occur twice: prior to starting either programme of rehabilitation and again after the discharge assessment on completion of the assigned programme.

### Non-clinical study outcomes

#### Web-usage audit for the internet-based programme

Weekly and total web usage statistics for patients assigned to the web-based programme will be monitored as will the number and type of any technical problems that the project team or participants encounter.

#### Uptake and drop out

We will monitor the number of patients with COPD who are eligible to partake in the study as well as the number of those people who proceed to consent. We will also monitor the number of participants who start and complete rehabilitation in both treatment groups as well as the number of people who drop out. We will record how long it takes participants to complete the web-based programme and for those who drop out, a record of how soon they drop out and how much of the programme they have completed. This will enable us to identify if there is a particular point at which more people are likely to give up. Any serious adverse events will be reported to the sponsor and patients’ ability to exercise safely will be monitored.

#### Sample size estimation and recruitment target

The study will aim to recruit as many suitable patients as are referred to the PR service within the operational phase. One of the main objectives of this feasibility study is to provide data on recruitment and to enable an accurate estimation of sample size for a planned RCT. Based on calculations from previous studies carried out in the PR service, we anticipate a recruitment rate of around 100 patients during our operational phase. This feasibility study will enable us to estimate the required sample size for the subsequent RCT based on a realistic recruitment strategy.

#### Randomisation

Participants will be randomised into one of two groups: the intervention group who will take part in the web-based PR programme and the standard care group who will undertake conventional PR at the site they were referred to. Randomisation to treatment group allocation will be a 1:1 ratio to either group and will use internet based ‘Sealed Envelope’ randomisation codes where treatment group allocation is sent by automated email to the research physiotherapist.

#### Quantitative data analysis

As this is a feasibility study, it is not appropriate at this stage to have sufficient statistical power to detect pre to post rehabilitation or between treatment group changes. Therefore for the quantitative data, analysis will primarily be descriptive that is, estimation of means and SDs, proportion of patients eligible/willing to participate in the study. Primary outcome data (ie, the ISWT) will be used to estimate a sample size calculation and non-inferiority margin for a RCT. We will also collect data on drop out and completion rates for both programmes.

Data will be entered and stored on a secure web-based system, REDCAP, which has discrepancy management features. Data can be transferred from REDCAP to the Statistical Package for the Social Sciences (SPSS) for statistical analysis.

All patient information that is collected during the course of the research will be kept strictly confidential. With the patient's permission, their general practitioner (GP) will be informed of their participation in the study and that they may be contacted for relevant reports. Any information about the patient which leaves the hospital will have their name and address removed. Participants will not be identified in any subsequent written material. Results will be reported in such a way that completely preserves confidentiality.

### Health economic evaluation

As part of the feasibility study, we aim to test the methods to be used in the proposed economic evaluation of the subsequent RCT. The perspective of the economic analysis will be that of the NHS and personal social service. Costs to be obtained will include the costs of providing web-based and the conventional rehabilitation programme. We will also test the validity and feasibility of administering the ‘Patient Cost Questionnaire’^24^ as well as the EQ-5D-5L for any future economic evaluation. The economic analysis will aim to examine the response rates, sample size calculation and other necessary information to calculate the cost savings for future economic evaluation.

### Qualitative research

The aim of the qualitative research is to identify possible barriers and facilitators to participation in a trial and in the web-based PR programme, in order to maximise chances of avoiding recruitment and retention problems during the eventual RCT and to help plan qualitative work that can contribute to a prospective process evaluation during the eventual trial.

We will invite patients to take part in a semistructured interview following their discharge assessment. We aim to conduct the interviews with a purposive sample of a wide range of participants including those who took part in the web-based rehabilitation programme as well as, if possible, with those people who dropped out.

The topic guide has been designed by a health psychologist in collaboration with our departmental PPI Advisory group to cover views of recruitment, randomisation, ongoing participation, completing the battery of assessments and any factors that may help or hinder participation in research and/or doing the rehabilitation programme such as support from family members etc. The interviews will be conducted by a health psychologist experienced in conducting qualitative research and will take place either at the hospital or in the patient's home as preferred.

#### Qualitative data analysis

Analysis will be via techniques based on a grounded theory approach.^25^ This is a data driven approach in which the researcher attempts to use the data to build a theory, while doing everything possible to ‘test’ the theory in the process. In the context of this feasibility study it would mean building a set of ideas about what could help, or get in the way of optimal recruitment and retention, with ways of testing emerging understanding as the qualitative component proceeds.

#### Qualitative methods

Analysis will involve audio recording of interviews (with informed consent) and verbatim transcription, then a team approach to a process known as constant comparison in which scrutiny of a small number of divergent transcripts gives rise to an initial coding frame (a set of categories of barriers and facilitators, for instance). The initial coding frame is then applied to subsequent transcripts, and if new data contradict the coding frame, the latter is revised and further data collection will confirm or disconfirm the model. At the end of this process the researcher has a coding frame that ‘fits’ the data (rather than trying to make the data ‘fit’ the coding frame). The findings would therefore be a set of issues that can help/hinder recruitment and retention, that could inform a subsequent trial.

### Ethics and dissemination

The findings from this feasibility study, particularly in relation to patient preferences and acceptability, will inform the design of any subsequent RCT. In the longer term we will seek to implement the findings of the RCT into daily practice and locally we will share the results of the feasibility study with local primary and secondary care interface groups, such as commissioning teams and service managers. We also aim to invite a local COPD commissioner to be a co-applicant for the full RCT and to sit on the study steering group. The steering group meets every 6 months to audit the trial conduct.

We have a number of international and national visitors who come to share our practice which we anticipate will stimulate interest in the study findings. We will be able to use the internet to widely publicise the findings of our work and promote interest in the internet-based programme.

We have a Pulmonary and Cardiac Rehabilitation PPI Advisory group whose members are current and ex-pulmonary and CR patients or interested members of the public who have participated in previous research trials. A strategy for disseminating the results will be coordinated through this group and the study steering group.

The results of this feasibility study will be presented at appropriate national, international and regional respiratory and physiotherapy conferences, local study days in primary and secondary care as well as through peer reviewed journals.
